# Social Isolation Induces Changes in the Monoaminergic Signalling in the Rat Medial Prefrontal Cortex

**DOI:** 10.3390/cells13121043

**Published:** 2024-06-17

**Authors:** Vivien Csikós, Fanni Dóra, Tamás Láng, Luca Darai, Vivien Szendi, Attila Tóth, Melinda Cservenák, Arpád Dobolyi

**Affiliations:** 1 Laboratory of Molecular and Systems Neurobiology, Department of Physiology and Neurobiology, Eötvös Loránd University, 1117 Budapest, Hungary; 2 Laboratory of Neuromorphology, Department of Anatomy, Histology and Embryology, Semmelweis University, 1094 Budapest, Hungary; 3 In Vivo Electrophysiology Research Group, Department of Physiology and Neurobiology, Eötvös Loránd University, 1117 Budapest, Hungary

**Keywords:** social behaviour, RNA-seq transcriptomics, anxiety and depression, dopaminergic system, serotonin receptor, dynorphin

## Abstract

(1) Background: The effects of short-term social isolation during adulthood have not yet been fully established in rats behaviourally, and not at all transcriptomically in the medial prefrontal cortex (mPFC). (2) Methods: We measured the behavioural effects of housing adult male rats in pairs or alone for 10 days. We also used RNA sequencing to measure the accompanying gene expression alterations in the mPFC of male rats. (3) Results: The isolated animals exhibited reduced sociability and social novelty preference, but increased social interaction. There was no change in their aggression, anxiety, or depression-like activity. Transcriptomic analysis revealed a differential expression of 46 genes between the groups. The KEGG pathway analysis showed that differentially expressed genes are involved in neuroactive ligand-receptor interactions, particularly in the dopaminergic and peptidergic systems, and addiction. Subsequent validation confirmed the decreased level of three altered genes: regulator of G protein signalling 9 (Rgs9), serotonin receptor 2c (Htr2c), and Prodynorphin (Pdyn), which are involved in dopaminergic, serotonergic, and peptidergic function, respectively. Antagonizing Htr2c confirmed its role in social novelty discrimination. (4) Conclusions: Social homeostatic regulations include monoaminergic and peptidergic systems of the mPFC.

## 1. Introduction

In social species, interactions between conspecifics are essential for the development and maintenance of normal behaviour and physiology [1,2]. Therefore, given the opportunity, individuals tend to spend time together. Rats, which are highly social animals with behavioural and physiological similarities to humans [3,4], provide a valuable model for studying the effects of social isolation [5]. Rats housed together tend to huddle and sleep in physical contact with each other. When awake, they prefer the stay in the vicinity of their conspecifics. If a new, unfamiliar rat is introduced, they will explore the unfamiliar animal [6]. When physical contact is allowed, they engage in various forms of social interaction. They sniff each other, perform allogrooming with their forepaws, and climb on top of each other. In addition to these affiliative behaviours, they may also exhibit sexual, aggressive, play, or caring behaviours depending on their age and sex [7]. The significance of social interactions is evident by the issues that arise due to social isolation, which is characterized by the absence of social contact. Social isolation has been recognized as a significant stressor with adverse consequences [5,8]. Adult rodents that were raised with partial maternal deprivation exhibit various behavioural dysfunctions, including social deficits, working memory impairment, and anxiety-like behaviours [9]. Post-weaning isolation can also alter their behaviour, as demonstrated by schizophrenia-like symptoms [10], increased aggression towards conspecifics [11,12,13], fear conditioning [14], and altered addictive behaviour [15]. Long-term adult isolation, which does not affect developmental processes as maternal deprivation does, has also been found to induce an aversive state, as evidenced by increased anxiety-like behaviour and fear responses [16,17], reduced exploration of novel environments, decreased social interaction [18], and elevated aggression [19]. In addition to these behavioural changes, the emotional well-being of rodents is also affected [20]. Long-term (4–6 weeks) adult social isolation is a model of depression [21,22,23,24] and has also been linked to alterations in cognitive functioning [25,26]. Rats subjected to prolonged social isolation exhibit deficits in learning and memory tasks, including spatial memory and object recognition [27].

The medial prefrontal cortex (mPFC) has been shown to regulate social interactions among rodents. The mPFC processes and integrates social information, enabling rats to recognize and respond to conspecifics appropriately. The mPFC also plays a crucial role in social cognition, such as recognizing social familiarity, forming social memories, and encoding social hierarchy [28,29,30]. Social isolation-induced disruption of mPFC function is associated with various behavioural changes in rats [31]. The mPFC has been implicated in increased anxiety- and depression-like behaviour, reduced sociability, and deficits in social memory and recognition characteristics of rats subjected to long-term social isolation [17,32]. There is also abundant evidence that altered mPFC activity can lead to suppression of the reward system resulting in depression-like behaviour [33]. This is known to develop following early-age social deprivation or long-term social isolation in adult rodents. Research has demonstrated that social isolation can cause structural and functional changes in the mPFC of rats [34,35]. Prolonged social isolation can result in modification in dendritic morphology, synaptic connectivity, and neurotransmitter receptor expression within the mPFC [29]. These changes are thought to affect the processing of social information and emotional regulation, leading to behavioural changes observed in socially isolated rats. Based on the role of mPFC in the behavioural alterations following long-term social isolation, it is possible that the area also participates in the behavioural changes, which take place after short-term social isolation. A shorter social deprivation does not lead to symptoms of neuropsychiatric disorders, but it can still be difficult to tolerate [36]. In rats and mice, social isolation for a few hours to days led to gradually increased social interactions, called social rebound [37], suggesting that social interactions have a homeostatic regulation [38,39].

Neurotransmitter systems, such as dopamine (DA), serotonin, gamma-aminobutyric acid (GABA), and neuropeptides, play crucial roles in the functioning of the mPFC [29,40]. Social interactions are rewarding for mice and rats. Indeed, dopaminergic neurons in the ventral tegmental area (VTA) are activated by social interaction even with a partner of the same sex [41]. The VTA DA neurons project to the mPFC, and the reward value of a partner reinforces social motivation affecting the mPFC decision-making systems for further social interactions [41,42]. Serotonergic signalling has also been implicated in modulating social behaviour. Alterations in serotonin levels have been linked to changes in the mPFC induced by social isolation [28]. In addition, GABA may also be involved in monoaminergic alterations in the mPFC following social isolation [43] and social play in relation to striatal connections [44] as well as aging and stress [45].

Thus, social isolation can disrupt the balance of mPFC neurotransmitters, which may contribute to the observed behavioural alterations [40]. In addition, social behaviour is also influenced by genomic mechanisms [46,47]. Changes in the mPFC related to social isolation likely involve changes in gene expression. Social isolation from weaning has been shown to lead to altered gene expression in the mPFC in mice [11,15,48] and rats [10] as revealed by RNA sequencing (RNA-seq). In rats, the effect of postweaning adolescent social deprivation has also been examined with microarray technique [49] and proteomics, confirming altered gene expression in the mPFC [14]. The impact of prolonged adult isolation has also been examined in the mPFC through RNA-seq [22] and proteomics in relation to the development of depression-like behaviour [50]. These studies all utilized different omics approaches to reveal gene-expression changes following early life or long-term social isolation. However, behavioural alterations have been established by shorter social isolation, too, as described above. Such shorter social isolation has been demonstrated to lead to gene-expressional alterations in the zebra finch brain [51] and also in the rat medial amygdala [52] but not in the mPFC. Therefore, in this study, we aimed to establish the behavioural consequences of 10 days of social isolation in adult rats and use RNA-seq to determine the accompanying gene-expression changes in the mPFC. Bioinformatics analysis, including gene-set enrichment and pathway analyses, revealed alterations in dopaminergic, serotonergic, and peptidergic systems due to social isolation, of which 1-1 representative was RT-qPCR validated and characterized as far as the localization of their expression. Furthermore, the role of the serotonin receptor 2C (Htr2c) was investigated through the determination of the behavioural effects of chronic brain infusion of a Htr2c antagonist, agomelatine [53].

## 2. Materials and Methods

### 2.1. Animals

The Workplace Animal Welfare Committee of the National Scientific Ethical Committee on Animal Experimentation of Eötvös Loránd University, Budapest, gave approval to this study with protocol code PE/EA/568-7/2021. The study involving rats was carried out in accordance with the guidelines of the Animal Hygiene and Food Control Department, Ministry of Agriculture, Hungary (40/2013), which comply with EU Directive 2010/63/EU for animal experiments. A total of 63 adult Wistar rats from Charles Rivers Laboratories, Hungary were used in this study. Of these, 15 + 15 were used for identification of behaviour following isolation, 12 for dissection for RNA-seq, 10 for RT-qPCR validation, and 10 for behavioural analysis followed by Htr2c antagonist injection. The animals were kept under standard laboratory conditions with a temperature of 23  ±  1 C and humidity of 50–60%. Rats were subjected to a 12-h light and 12-h dark cycle, with lights on at 6:00 AM. The experimental procedures were conducted in a separate room where the animals remained for the entire experiment. The animals had access to feed (SM R/M-H, 1534-00; Ssniff, Soest, Germany) and drinking water *ad libitum*. The initial body weight of the animals was 289 ± 23 g at the beginning, and 301 ± 27 g at the end of the experiments. The abnormal behaviour of the rats was never observed in the study. For surgeries and dissections, the animals were anaesthetized with an intraperitoneal injection of an anaesthetic mix containing 0.4 mL/300 g body weight ketamine (50 mg/mL) and 0.2 mL/300 g body weight xylazine (20 mg/mL).

### 2.2. Behavioural Tests

#### 2.2.1. Isolation of Animals and Study Design

Fifteen adult male animals, aged 3 months and weighing 300–330 g, were subjected to 10 days of social isolation, while a control group of fifteen conspecifics remained housed in pairs. The cages were placed in the same room of the animal facility throughout the isolation procedure.

**Figure 1:**
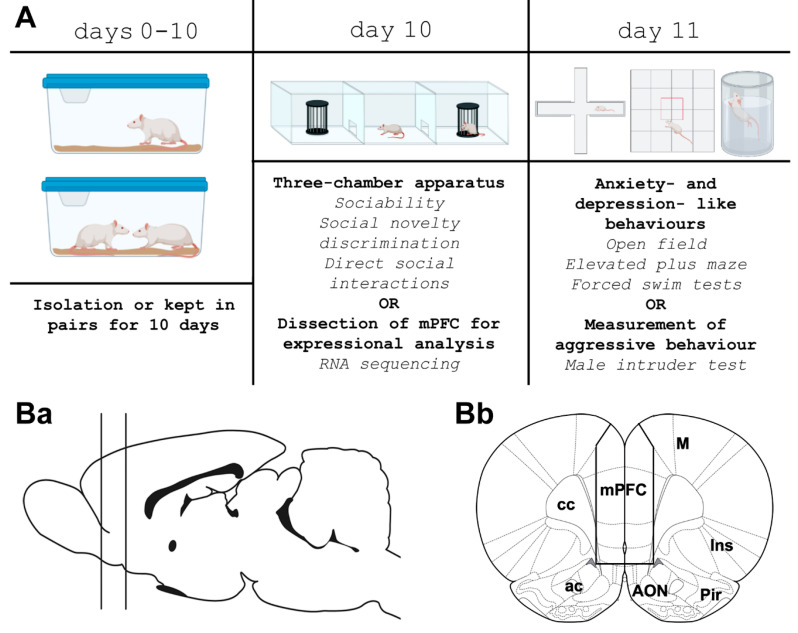
Design of the social-isolation experiment. (**A**): Rats were isolated or kept in pairs for 10 days, when either a three-chamber test or dissection of the medial prefrontal cortex (mPFC) took place. The animals that were not sacrificed were kept isolated (or paired) overnight and were further tested for anxiety- and depression-like behaviours the following day. (**B**): Dissection of mPFC samples shown in drawings based on the rat brain atlas [55]. (**Ba**): The position of the coronal section cut as a first step of mPFC dissection is shown by the 2 vertical lines on the schematic side view of the brain. (**Bb**): The mPFC tissue samples were obtained from the coronal section by cuts at the solid lines. Additional abbreviations: ac—anterior commissure, AON—anterior olfactory nucleus, cc—corpus callosum, Ins—insular cortex, M—motor cortex, Pir—piriform cortex.

In order to be able to correlate behaviour and transcriptomics, the behavioural tests were compounded into 2 days. On the 10th day of isolation, both isolated and control animals were habituated to the test apparatus for 5 min each. The first battery of behavioural tests was conducted on the 10th day of isolation, which included tests for sociability, social novelty discrimination, and direct social interactions for 15 animals (Figure 1A). On the 11th day of isolation, the animals were tested again but both groups were divided into 2 subgroups because all tests cannot be performed on the same animal. Thus, 8 randomly selected animals per group performed open-field, elevated-plus-maze, and forced-swim tests to measure locomotion, anxiety, and depression-like behaviours. Additionally, the male-intruder test was conducted on the same day with the other 7 animals per group to quantify their aggressive behaviours. The animals were videotaped throughout the behavioural tests using an SJCAM SJ4000 FULLHD action camera (Shenzen, China) at a 60 Lux lightening. The videotapes were analysed using Solomon Coder Software version 17.03.22 accessed on 22 March 2022 [54] (https://solomon.andraspeter.com/) for direct social-interaction, male-intruder, elevated-plus-maze, and forced-swim tests. The SMART Video Tracking Software v3.0 (Panlab Harvard Apparatus, Barcelona, Spain) was used for open-field, sociability, and social novelty discrimination tests. Following each test, the animals were promptly returned to their home cages, and the apparatus was cleaned with a 70% ethanol solution to eliminate odour trails.

#### 2.2.2. Sociability and Social Novelty Discrimination

The experiment was conducted using a three-chamber apparatus consisting of three interconnected chambers, each measuring 40 × 80 cm, connected by doors, as per the protocol [56]. The two lateral chambers contained small cages, one of which housed a conspecific while the other remained unoccupied. This allowed the animals to smell, hear, and see each other during the session but prevented direct contact. The subject was free to move between the chambers throughout the 10-min test period. The initial test of sociability was immediately followed by the social novelty discrimination test. This test began when an unfamiliar animal was placed in the previously empty small cage and lasted for 10 min. The data were analysed using a two-way ANOVA with the time spent in different compartments and the groups of animals (isolated vs. paired) as the two parameters, followed by Holm-Šídák’s multiple comparison tests.

#### 2.2.3. Social-Interaction Test

During the direct social test, a previously habituated subject and a familiar partner animal were placed in an open-field arena measuring 40 × 80 cm. The animals were free to move and interact with each other, and 10 min of footage were recorded. For affiliative behaviour, body sniffing (the nose of the test animal is close to the non-anogenital region of the other animal), anogenital sniffing (the nose of the test animal is close to the anogenital region of the other animal), social grooming (the test animal gently nibbles or licks the fur of a conspecific, often with the aid of its forepaws), and mounting (the test animal walks over the other animal) were coded. Additionally, the analysis extended to include non-affiliative social behaviour, such as chasing (the test animal approaches the partner) and side-to-side contacts (where the animals were in contact with each other’s sides), as well as passive social interactions (instances where the partner animal displayed the aforementioned affiliative social behavioural elements while the test animal did not exhibit any social behaviour). The data were analysed using a two-way ANOVA with the behavioural elements and groups of animals as the two parameters, followed by Šídák’s multiple comparison tests.

#### 2.2.4. Male-Intruder Test

At the end of the second day of testing, a male intruder weighting 20–40 g less than the resident animal test was conducted. The test animal was left in its home cage while an unfamiliar male rat was introduced, resulting in an aggressive response. To assess aggression, dominant mounting, fighting, and biting were coded from 10 min of video footage. The data were analysed using a two-way ANOVA with the behavioural elements and animal groups as parameters, followed by Holm-Šídák’s multiple comparison tests.

#### 2.2.5. Open-Field Test

To measure locomotor activity and assess anxiety-like behaviour, the animals were observed in an open-field arena (40 × 80 cm) and their movements were recorded for 10 min. The arena was divided into three zones: the periphery, the middle, and the centre. The number of entries into each zone was evaluated using the SMART system. The data were analysed using a two-way ANOVA with the behavioural elements and animal groups as the two parameters, followed by Holm-Šídák’s multiple comparison tests.

#### 2.2.6. Elevated-Plus-Maze Test

To assess anxiety and depression-like behaviours, rats were tested in the elevated plus maze (EPM) for 10 min to evaluate their anxiety levels. The EPM apparatus consisted of two open arms (40 × 6 cm) and two closed arms of similar size, connected by a central platform (6 × 6 cm), and was elevated 40 cm above the floor. At the start of the session, each rat was placed in the centre of the maze, facing one of the open arms, and its behaviour was recorded for 10 min. To compare the control and treated groups, we used a two-way ANOVA with entries to different compartments and animal groups as parameters

#### 2.2.7. Forced-Swim Test

At the end of the second day of testing, a forced-swim test (FST) was conducted. The test began one hour after the elevated-plus-maze (EPM) test to allow the animals to rest. Plastic cylindrical tanks measuring 50 cm in height and 25 cm in diameter were used, with the water temperature maintained at 24 °C. The test lasted for six minutes. Following the FST, the animals were dried with a towel and returned to their home cages. The videos were evaluated for swimming, climbing, and floating behaviours. To compare the two groups (isolated vs. paired and antagonist- vs. vehicle injected), a two-way ANOVA was used with the behavioural elements and animal groups as the two parameters.

### 2.3. Microdissection of Medial Prefrontal Cortex

For RNA-seq and quantitative reverse transcription PCR (RT-qPCR), we microdissected the medial prefrontal cortex from 12 rats for RNA sequencing and from 10 rats for RT-qPCR. The whole medial prefrontal cortex was dissected with all of its parts, including the infralimbic and prefrontal cortices despite the differences in their structure and function. Half of the animals were isolated, while the other half were paired for 10 days before dissection. The animals were anesthetized with an intraperitoneal injection of an anaesthetic mix containing 0.4 mL/300 g body weight ketamine (50 mg/mL) and 0.2 mL/300 g body weight xylazine (20 mg/mL) before brain dissection. Coronal brain sections (2 mm thick) were subsequently prepared using a razor blade. The anteroposterior levels were determined using the anterior level of the optic chiasm (bregma level: +0.3 mm). To dissect the mPFC sample, a coronal section was first cut between anteroposterior bregma levels 4.3 and 2.3 mm as described previously [55]. The ventral 1.5 mm of this section, which contains the anterior olfactory nucleus, was subsequently removed (Figure 1Ba). Then, vertical cuts were made 1 mm lateral to the midline to include the mPFC from both sides of the brain (Figure 1Bb). The dissected tissue samples were promptly frozen in Eppendorf tubes on dry ice and stored at −80 °C.

### 2.4. RNA Sequencing

Total RNA was extracted followed by processing for quality control. RNA sequencing (RNA-seq) was performed on 11 prefrontal cortical tissue samples from rats (5 isolated and 6 pair-kept animals; one isolated sample was excluded due to low RNA quality) using the HiSeq2000 platform (Illumina, San Diego, CA, USA) by Macrogen Inc. (Seoul, Republic of Korea). Library construction (TruSeq Stranded Total RNA Kit, Illumina) was performed using a 50-bp single-end sequencing strategy. The library’s size, concentration, and integrity were verified using an Agilent 2100 Bioanalyzer (Agilent Technologies, Santa Clara, CA, USA). After sequencing, the read quality was checked using the FASTQC tool. Low-quality reads and adapter sequences were removed using Trimmomatic [57]. The filtered data was then mapped to the reference genome of Rattus Norvegicus (mRatBN7.2) downloaded from the Ensembl database (https://ftp.ensembl.org/pub/release-102/fasta/rattus_norvegicus/dna/) accessed on 1 November 2021 using HISAT2 (v2.1.0) [58]. Aligned reads were assembled into transcripts and their abundance was estimated using StringTie, John Hopkins University, Baltimore, USA (v1.3.4) [59]. The gene expression levels between groups were compared using FPKM (fragments per kilobase of exon per million fragments mapped), which measures the relative abundance of genes.

### 2.5. Bioinformatics

Differentially expressed genes (DEGs) were identified using Cuffdiff version 2.2.1, a separate program in Cufflinks, Seattle, Washington, USA [60]. Cuffdiff calculated expression in two or more samples and tested the statistical significance of each observed change. Genes with a *p*-value < 0.0001, *q*-value < 0.05, and log2-fold change > ±0.5 were identified as differentially expressed. The DEGs were visualized in a volcano plot created with the R package ggplot2 (v3.3.5).

The study utilized the Database for Annotation, Visualization, and Integrated Discovery (DAVID) database [61] to conduct gene ontology (GO) and the Kyoto Encyclopedia of Genes and Genomes (KEGG) pathway mapping analysis on differentially expressed genes (DEGs) between paired and isolated groups. The potential targets of DEGs were analysed using GO, while their enriched pathways were analysed using the KEGG program. The analysis of gene ontology (GO) included terms related to biological processes, molecular functions, and cellular components. A *p*-value of less than 0.05 was considered statistically significant. For the KEGG pathway enrichment analysis, pathways with a *p*-value of less than 0.05 were considered enriched. Protein-protein interaction (PPI) networks were constructed and analysed using the Search Tool for the Retrieval of Interacting Genes/Proteins (STRING) version 11.5, Zurich, Switzerland and Cytoscape, version 3.8.2, Cytoscape consortium, Bethesda, MD, USA.

### 2.6. Quantitative RT-PCR

To confirm the RNA-seq data, a subset of differentially expressed genes was validated using quantitative real-time PCR (qRT-PCR). The procedure was carried out as described previously [62]. Briefly, total RNA was isolated from approximately 20 mg of frozen postmortem brain tissue using TRIzol reagent (Invitrogen, Carlsbad, CA, USA) as the lysis buffer combined with the RNeasy Mini kit (Qiagen, Stockach, Germany) following the manufacturer’s instructions. The RNA quality and quantity were assessed using a NanoDrop ND-1000 Spectrophotometer (Thermo Fisher Scientific, Waltham, MA, USA). Only samples with an A260/A280 ratio between 1.8 and 2.1 were used in subsequent experiments. The RNA concentration was adjusted to 2 µg/µL with RNase-free water and treated with Amplification Grade DNase I (Invitrogen, Carlsbad, CA, USA). The RNA was isolated and then reverse transcribed into cDNA using the SuperScript II Reverse Transcriptase Kit (Invitrogen, Carlsbad, CA, USA). The resulting cDNA was diluted 10-fold and 2.5 µL was used as a template for PCR, which was performed in duplicate using SYBR Green dye (Sigma, St. Louis, MO, USA). The PCR reactions were performed using iTaq DNA polymerase (Bio-Rad Laboratories, Hercules, CA, USA) on a CFX-96 C1000 Touch Real-Time System (Bio-Rad Laboratories, Hercules, CA, USA) with a total volume of 12.5 µL. The reactions were carried out under the following conditions: 95 °C for 3 min, followed by 35 cycles of 95 °C for 0.5 min, 60 °C for 0.5 min, and 72 °C for 1 min. A melting curve was performed at the end of the amplification cycles to verify the specificity of the PCR products. The PCR primers were synthesized by Integrated DNA Technologies, Inc. (IDT, Coralville, IA, USA) and used at a final concentration of 300 nM. The housekeeping genes ACTB, GAPDH, and LDHA were used as controls, and the relative gene expression values were calculated from their averages using the 2ΔΔCt method.

### 2.7. Htr2c Antagonist Treatment

Agomelatine (Merck A1362), a Htr2c antagonist, was administered via osmotic minipump delivery (ALZET Micro-Osmotic Pump model 2004, Durect™, Cupertino, CA, USA) continuously into the lateral ventricle. The drug and vehicle were delivered to the treated and control groups of animals, respectively. Five animals were treated with Agomelatine, for them, the pump was filled with 1 mg/mL Agomelatine dissolved in 5% dimethyl-sulfoxide (DMSO; Merck 20-139) while the other five control animals received a 5% DMSO solution. The pumps had a maximum capacity for 15 days of perfusion, delivering at a rate of 0.5 µL per hour. Thus, a daily quantity of 0.08 mg/kg body weight was injected into the 300 g animals. Both groups were housed in pairs throughout the experiment, except for the first day following surgery when they were housed individually.

Osmotic minipump implantation was performed as described previously [63]. Briefly, rats were anaesthetized with an intraperitoneal injection of an anaesthetic mix containing 0.4 mL/300 g body weight ketamine (50 mg/mL) and 0.2 mL/300 g body weight xylazine (20 mg/mL). Subsequently, the rats were placed in a stereotaxic apparatus, the skin was cut, and a hole of about 1 mm in diameter was drilled in the left side of the skull above the lateral ventricle at the following coordinates: −0.5 mm anteroposterior to the bregma; 1.4 mm lateral to the bregma. Cannulas were inserted into the lateral ventricle (3.6 mm ventral to the surface of the brain) and secured to the skull with cranial plastic cement. The pumps were placed subcutaneously at the back of the animals. After surgery, the animals were administered Tardomyocel^®^ comp. Antibiotics III (0.1 mL/kg) subcutaneously for 3 days to prevent infection. The osmotic minipumps continuously released Agomelatine for a maximum of 15 days, during which time behavioural tests were conducted on the animals at 10 and 11 days after implantation of the pump.

### 2.8. Statistical Analysis

Statistical analyses, including tests of normality and a follow-up ANOVA, were conducted using GraphPad Prism (GraphPad Software, version 9.0, LLC) if more than 2 groups were analysed, and with Student’s test for 2-group comparisons. Statistical significance was determined at *p* < 0.05, with * indicating 0.010 < *p* < 0.050, ** indicating 0.001 < *p* < 0.010, and *** indicating *p* < 0.001. All values are expressed as mean ± s.e.m.

## 3. Results

### 3.1. Behavioural Effects of Isolation

#### 3.1.1. Sociability

**Figure 2:**
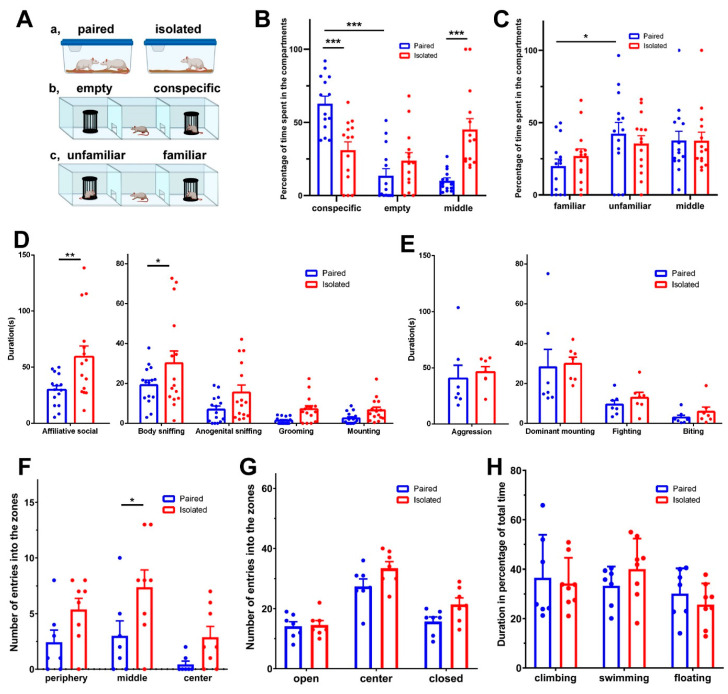
The effect of social isolation on the behaviour of rats. The blue boxes represent data from rats previously kept in pairs, while the red boxes represent data from previously isolated rats. Individual data points are represented by dots. (**A**): Schematic drawing of rats kept in pairs or housed individually (a), as well as the three-chamber apparatus used to measure sociability (b) and social novelty discrimination (c). (**B**): The result of the measurement of sociability. The percentage of the total testing time spent in the compartments containing the conspecific, the empty, and the middle compartments. Previously pair-kept animals spent significantly more time in the compartment with the conspecific than the isolated animals. In turn, they spent less time in the middle compartment (***: *p* < 0.001). (**C**): The result of the measurement of social novelty discrimination. After introducing an unfamiliar animal to the test rat in an empty compartment, there was no difference in the time spent in any of the compartments between the previously paired and isolated animals (*: *p* < 0.05). (**D**): The results of the direct social-interaction test. The isolated animals spent more time exhibiting affiliative behaviours (**: *p* < 0.01). Although all forms of affiliative social behaviour showed a tendency to increase, only body sniffing was significantly elevated (*: *p* < 0.05). (**E**): The male-intruder test results showed no difference in aggressive behaviour between the two groups. (**F**): In the open-field test, the previously isolated animals showed significantly more entries into the middle zones (*: *p* < 0.05). (**G**): The elevated-plus-maze test results showed no significant difference in the number of entries into the open and closed arms of the test apparatus between the two groups. However, isolated animals showed a tendency towards an elevated number of entries into the closed arm. (**H**) The results of the forced-swim test indicated that the two groups did not differ in the time spent climbing, swimming, or floating.

Isolating the animals for 10 days altered their preference in the three-chamber test (Figure 2A). The paired animals behaved differently than the isolated animals (Two-way ANOVA, F(2,81) = 16.48, *p* < 0.001, partial η^2^ = 0.29). The animals kept in pairs spent 62.8 ± 5.1% of the time in the compartment of the three-chamber apparatus where the conspecific was located, and only 13.5 ± 4.9% in the empty compartment, which is a significant difference (Holm-Sidak’s multiple comparison test, *t* = 6.578, *p* < 0.001). In contrast, the animals that were isolated spent significantly less time in the compartment with the conspecific (32.3 ± 5.5%, Holm-Sidak’s multiple comparison test, *t* = 4.146, *p* < 0.001). Furthermore, they spent 23.2 ± 5.2% in the empty cage, which is not significantly less than the time they spent in the compartment of the conspecific. In turn, the isolated animals spent most of their time in the middle compartment (Figure 2B).

#### 3.1.2. Social Preference

In the social novelty discrimination test, a new conspecific was introduced into the previously empty cage of the three-chamber apparatus, providing the test animals with an opportunity to distinguish between the already familiar and an unfamiliar animal (Figure 2A). The paired group of animals spent 19.9 ± 4.9% of the test period in the compartment of the familiar rat and 42.4 ± 7.8% in the compartment of the unfamiliar rat, indicating a significant difference between the amounts of time spent in the two compartments (Holm-Sidak’s multiple comparison test, *t* = 2.721, *p* < 0.05). In contrast, the isolated animals spent 26.6 ± 4.6% of their time in the compartment of the familiar rat. In the compartment containing the new, unfamiliar conspecific, the isolated group of animals spent 35.6 ± 5.0% of their time, which is not significantly different from the time spent in the compartment with the familiar animal (Figure 2C). Both groups spent the remaining time in the middle compartment of the apparatus.

#### 3.1.3. Affiliative Interactions

The affiliative behaviour of rats was explored through a direct social-interaction test in a familiar arena. The paired animals behaved differently than the isolated animals (Two-way ANOVA, F(1,84) = 5.93, *p* < 0.05, partial η^2^ = 0.07). Isolated rats displayed a longer duration of affiliative behaviour compared to paired rats (59.3 ± 9.7 vs. 29.8 ± 3.7 s; Sidak’s multiple comparison test, *t* = 2.77, *p* < 0.05). Further analysis revealed a significant increase in the duration of body sniffing (30.1 ± 6.2 vs. 19.2 ± 2.5 s; Sidak’s multiple comparison test, *t* = 3.29, *p* < 0.01). Although anogenital sniffing, grooming, and mounting did not reach statistical significance, these behaviours also showed an increasing trend in the isolated animals (Figure 2D). On the other hand, isolation had no effect on the duration of non-affiliative social behaviour (61.9 ± 9.6 vs. 68.7 ± 7.8 s). In turn, isolation affected not only the test animal but also their partner animals despite those animals not being separated previously. Although the behaviour of the partner animals did not change significantly, they displayed an increasing trend in the duration of affiliative behaviour when the subject animals were previously isolated (35.2 ± 8.5 vs. 12.9 ± 2.7 s), which we considered as passive social interaction from the point-of-view of the test animal.

#### 3.1.4. Aggressive Behaviour

The day after conducting a social-interaction test in an arena, a male-intruder test was performed in the home cage of the isolated and the pair-kept animals. During the test, a single home-cage animal was present in the cage and an unknown intruder animal was placed there. In this situation, the host animal displayed aggression towards the foreign intruder placed in its home cage. The animals that were previously isolated spent an average of 40.6 ± 11.7 s, while the paired animals spent 46.1 ± 5.0 s exhibiting aggressive behaviour. This suggests that isolation did not have a significant impact on aggressive behaviour (Two-way ANOVA, F(1,36) = 0.61, *p* > 0.05, partial η^2^ = 0.02). We also investigated specific aggressive behaviours, such as dominant mounting, fighting, and biting, but we did not observe any differences in these behaviours between the isolated and paired animals (Figure 2E).

#### 3.1.5. Locomotor Activity

The number of entries to the middle zone was significantly (unpaired *t*-test, *t* = 52.24, df = 13, Cohen’s d effect size = 1.16, *p* < 0.05) higher for the isolated animals than for the paired animals (periphery: 5.4 ± 1.0 vs. 2.4 ± 1.1 times; middle zone: 7.4 ± 1.6 vs. 3.0 ± 1.3 times; centre: 2.9 ± 1.0 vs. 0.4 ± 0.3 times), suggesting hyperlocomotion in the previously isolated group of animals (Figure 2F).

#### 3.1.6. Anxiety-like Behaviour

The anxiety-like behaviour of the animals was assessed using an elevated plus maze. The paired animals entered the open arms 14.1 ± 1.5 times and the closed arms 15.7 ± 1.6 times. The isolated animals entered the open arms 14.6 ± 1.5 times and the closed arms 21.4 ± 2.1 times. The closed-arm entry of isolated animals showed a tendency towards an increase (unpaired *t*-test, *t* = 2.14, df = 12, Cohen’s d effect size = 1.15, *p* < 0.1), indicating a potential anxiety-like phenotype (Figure 2G).

#### 3.1.7. Depression-like Behaviour

The depression-like behaviour of the experimental rats was evaluated using the forced-swim test. The paired animals exhibited 36.6 ± 6.5% climbing, 33.3 ± 2.9% swimming, and 30.1 ± 3.9% floating. The animals kept in isolation spent 34.3 ± 3.6% of their time climbing, 40.1 ± 4.3% swimming, and 25.7 ± 3.0% floating. There were no significant differences in any of the three measured behavioural elements between the two groups assessed by unpaired *t*-tests (Figure 2H).

### 3.2. Altered Gene Expression in the mPFC Due to Social Isolation

#### 3.2.1. RNA-Seq

**Figure 3:**
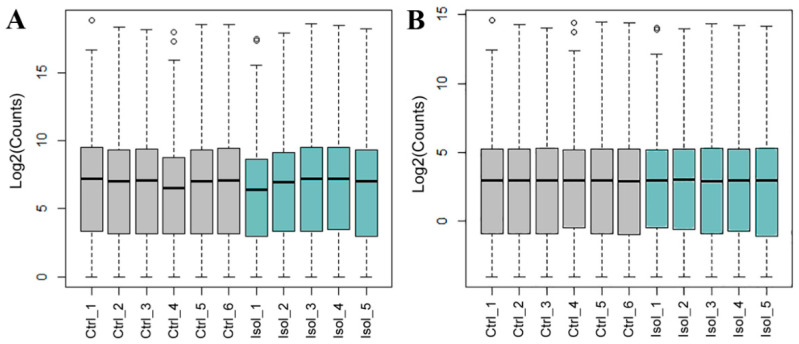
Quality control of RNA-Seq transcriptomic data. Box plots showing expression distribution for (**A**) filtered un-normalized and (**B**) normalized count data. The trimmed mean of m-values (TMM) method was used to correct the read-count differences between the samples. The median is represented by the inside lines, and the upper and lower quartiles are indicated by the top and bottom of the box, respectively. The graph displays the highest and lowest values through the whiskers, while the excluded outliers are represented by circles. The abbreviations ‘Ctrl’ and ‘Isol’ stand for ‘control’ and ‘isolated’, respectively. The numbers refer to the number of animals per group.

**Figure 4:**
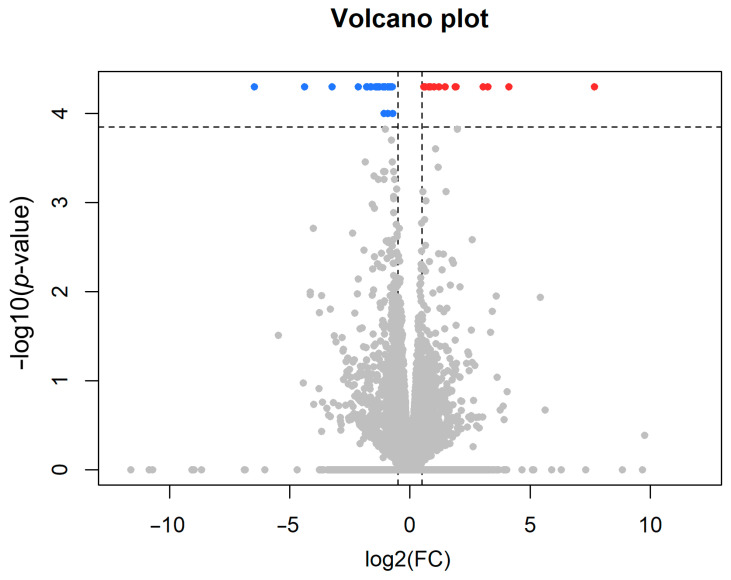
Representation of the altered genes in the sequencing experiment. The volcano plot displays genes that are up- and downregulated with RNA sequencing based on statistical significance versus magnitude of change. The thresholds were set at a *p*-value of <0.0001 and a log2-fold change of >0.5, respectively. The blue dots represent the downregulated genes, while the red dots represent the upregulated genes. Genes, which were neither up- nor downrgulated are shown in grey.

The RIN values were high for all 11 samples sequenced, ranging from 8.3 to 10. The 12th sample, an isolated one, was not sequenced due to its low RIN value (<6). The mapping percentages to the reference genome were consistently high across all samples (Figure 3). On average, 35.9 ± 1.0 Mb of total reads were generated per sample. The average mapping ratio with the reference genome was 97.1 ± 0.2%, while the average mapping ratio with genes was 47.0 ± 2.2%. Table 1 shows the number of total reads and their percentages. To eliminate the impact of non-biological factors on RNA-seq data, we conducted quality control (QC) on each raw file. Figure 2 displays the results of QC box plots before and after normalization. We identified 46 differentially expressed genes (DEGs) (Table 2), which were visualized in a volcano plot created using the R package ggplot2. The statistical significance threshold and fold change were set at a *p*-value of <0.0001 (*q*-value < 0.05) and a log2-fold change of >±0.5, respectively (Figure 4).

#### 3.2.2. Bioinformatics Analysis of DEGs

**Figure 5:**
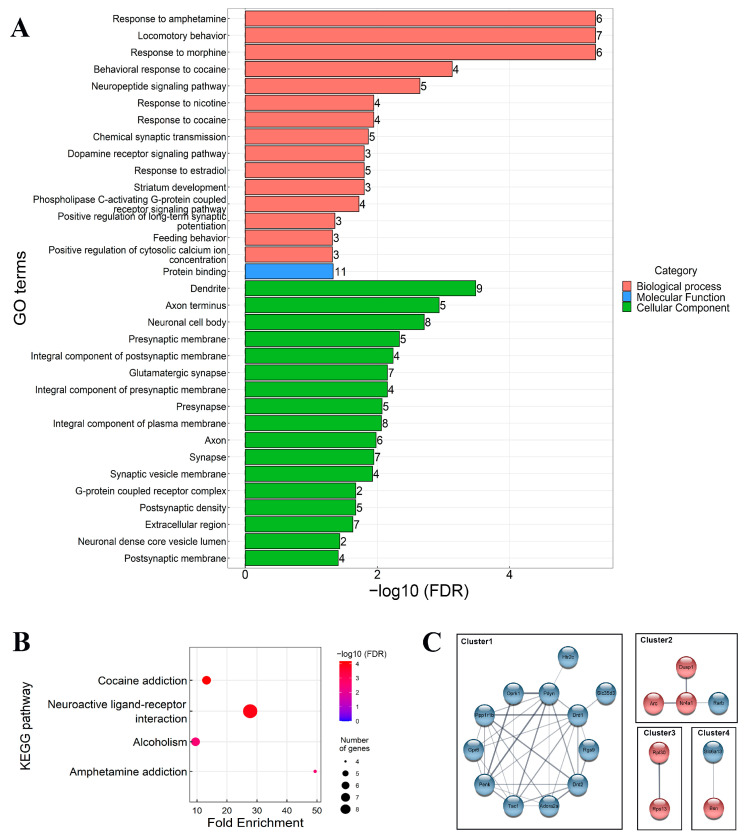
The visualization of bioinformatics results. (**A**): GO-terms significantly enriched (false discovery rate [FDR] < 0.05) in the isolated group of animals in the mPFC. The division of genes shows changes in their expression based on their biological function, molecular function, and location in the cell. The majority of variable genes were associated with addiction and located in synapses. (**B**): t plots were used to illustrate the distribution of gene sets among up- and downregulated differentially expressed genes from KEGG pathway analysis. The *x*-axis represents the gene ratio, which refers to the value of enrichment. The *y*-axis represents the pathway term. This is the ratio of DEGs annotated in the pathway to the total gene amount annotated in the pathway. The larger the value, the more significant the enrichment. The size of the circle indicates the DEG number associated with each significant pathway, while the colour indicates the adjusted *p*-value. The size of the circle indicates the DEG number associated with each significant pathway, while the colour indicates the adjusted *p*-value. A lower *p*-value indicates a more significant enrichment. (**C**): STRING displays the known and predicted protein-protein interactions of the different DEGs in our study. Associations are based on confidence and require a medium confidence (0.4) interaction score. We applied MCL network clustering (inflammation parameter: 3). Interactions in this study are described in terms of physical and functional associations. The differentially expressed genes (DEGs) are grouped into four distinct clusters. The blue circle represents the interaction network of downregulated hub genes, while the red circle indicates the interaction of upregulated hub genes.

To evaluate the function of the DEGs, we conducted Gene Ontology (GO) classification and KEGG functional enrichment using the DAVID online database. We visualized all enriched terms within categories (Figure 5A). Based on GO classification, the down- and upregulated genes are associated with monoaminergic and peptidergic biological functions. DEGs were primarily located in the dendrite, axon terminus, and neuronal cell body. The KEGG pathway analysis revealed that DEGs were enriched in different forms of addiction, as well as neuroactive ligand-receptor interaction (Figure 5B). Furthermore, STRING analysis showed that DEGs clustered around four distinct protein interaction clusters (Figure 5C). Pathway analysis of these clusters conducted through STRING highlighted essential genes grouped in Cluster 1. The cluster showed enrichment in positive regulation of dopamine and acetylcholine neurotransmission, neurotransmitter receptor activity, and behavioural patterns related to addiction and maternity.

#### 3.2.3. Validation of Selected Genes

**Figure 6:**
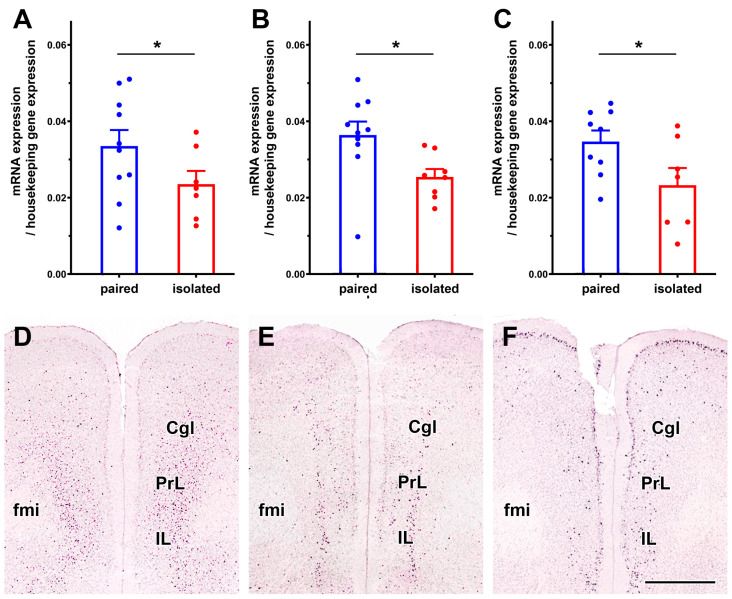
RT-qPCR validation of 3 genes with reduced levels in the isolated animals. (**A**): Rgs9: regulator of G protein signalling 9 (Rgs9). (**B**): serotonin receptor 2C (Htr2c). (**C**): Prodynorphin (Pdyn). The values for the paired group are shown in blue, while the values for the isolated group are shown in red. The validation demonstrated a significant decrease in all three cases (*: *p* < 0.05). The distribution of the genes in coronal sections of the mouse mPFC is illustrated in the pictures selected from the in situ hybridization database of the Allen Brain Atlas [64]. The expression sites of the genes are shown in black, as reported by Atlas. All three genes are expressed by a subclass of mPFC neurons. However, their distribution patterns differ: Rgs9 is ubiquitously distributed in the deep layers (**D**), Htr2 is most abundant in layer IV (**E**), while Pdyn expression has the highest density in superficial layer II (**F**). Scale bar = 1 mm. Magnification is the same for panels D-F. Abbreviations: Cgl—cingulate cortex, fmi—forceps minor, IL—infralimbic cortex, PrL—prelimbic cortex.

The expression of three genes, regulator of G protein signalling 9 (Rgs9), serotonin receptor 2c (Htr2c), and prodynorphin (Pdyn), involved in dopaminergic, serotonergic, and peptidergic function, respectively, was validated using RT-qPCR. All three genes exhibited decreased expression in the RNA-seq (Figure 6A–C), which was confirmed by the independent technique of RT-qPCR, as all three genes had reduced mRNA levels in the isolated group of animals (unpaired *t*-tests, df = 14, *p* < 0.05; t and Cohen’s d effect size = 2.32 and 1.19 for Rgs9, 2.51 and 1.23 for Htr2c, and 2.24 and 1.10 for Pdyn, respectively). The expression of these genes in the medial prefrontal cortex (mPFC) was validated by searching the Allan Brain Atlas database. In situ hybridization performed in mice confirmed that all three genes were expressed in the mPFC, but only a subset of neurons expressed them. The distribution of neurons expressing the three genes differed within the mPFC (Figure 6D–F). Rsg9 was most abundant in deep layers while also present in layers III but almost absent in layers I-II. Htr2c is expressed in layer VI, less abundantly in layer III, while some labelled cells are found in layer II as well. In contrast, Pdyn is expressed in layers II and III, less in layer V but is not expressed in layer VI.

### 3.3. Behavioural Analysis after Htr2c Antagonist Treatment

#### 3.3.1. Alterations in Social Behaviours

**Figure 7:**
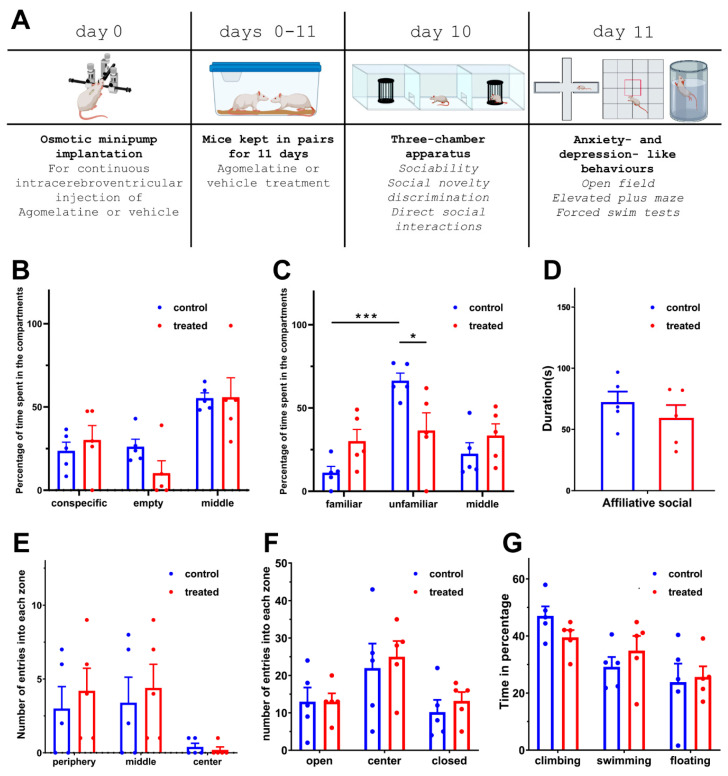
The effect of continuous intracerebroventricular agomelatine treatment on the behaviour of rats in the three-chamber apparatus. Data of individual animals are shown by the dots. (**A**): Experimental design. Animals were implanted with osmotic minipumps for continuous injection of agomelatine (or vehicle) into the cerebral ventricle. The rats were then isolated or pair kept for 11 days. Behavioural tests in a three-chamber apparatus were performed followed by tests of anxiety- and depression-like behaviour the following day. (**B**): Result of testing sociability. The percentage of the total testing time spent in the compartments containing the conspecific, the empty, and the middle compartments. Blue data show how much time the control animals spent in the compartments while red shows the data of agomelatine-treated animals. The time spent in any of the compartments did not differ between the control and the agomelatine-treated animals. (**C**): Social novelty discrimination. After introducing an animal unfamiliar to the test rat in the so far empty compartment, the control animals spent significantly more time in the compartment with the newly introduced unfamiliar animal than the treated rats (***: *p* < 0.001, *: *p* < 0.05). (**D**): The duration of time the animals spent with affiliative behavioural components while directly interacting with each other. (**E**): Open-field test. The number of entries into the different zones (periphery, middle, and centre) are shown in the isolated and pair-kept group of animals. (**F**): Elevated-plus-maze test. The number of entries into the open, centre, and closed compartments are shown. (**G**): Forced-swim test. The duration of time spent climbing, swimming, and floating is demonstrated for the isolated and pair-kept group of animals.

In this experiment, all animals were housed in pairs because we wanted to mimic the effect of reduced functioning of Htr2c in isolated animals by antagonizing the Htr2c in one group of animals with agomelatine while the other group received the vehicle into their lateral ventricle (Figure 7A). The sociability of the groups did not differ. The control animals spent 23.7 ± 5.2% of their time in the compartment with the conspecific and 26.1 ± 4.5% in the empty compartment. The animals that were treated with agomelatine spent 30.2 ± 8.7% of their time in the compartment with the conspecific and 10.3 ± 7.4% in the empty compartment (Figure 7B). These values were not statistically different from those of the control group (Two-way ANOVA, F(1,24) = 0.24, *p* > 0.05, partial η^2^ = 0.01).

During the social novelty discrimination test, a new and unfamiliar rat was introduced into the three-chamber apparatus. The test animals were given the option to stay in the compartment with the familiar rat or the new, unfamiliar one. The control animals demonstrated a significant preference for the unfamiliar rat, spending 66.4 ± 4.6% of their time in its compartment compared to 11.1 ± 3.8% in the compartment with the familiar rat (Holm-Sidak’s multiple comparison test, *t* = 5.61, *p* < 0.001). In contrast, the animals treated with the antagonist spent 30.1 ± 7.0% of their time in the compartment containing the familiar animal and 36.5 ± 10.6% of their time in the compartment of the new, unfamiliar rat, indicating no statistically significant preference for the novel unfamiliar animal in the same test (Figure 7C). Additionally, the time that the treated and control animals spent in the compartment with the unfamiliar animal was statistically different (Sidak’s multiple comparison test = 3.04, *p* < 0.05). Thus, the antagonist treatment decreased the preference for the novel unfamiliar rat.

During social-interaction tests in a familiar arena, the animals treated with a Htr2c antagonist did not demonstrate significant differences compared to control animals (Two-way ANOVA, F(1,24) = 0.05, *p* > 0.05, partial η^2^ = 0.01). The treated animals spent 59.4 ± 10.5 s with affiliative behaviour (social sniffing, grooming, mounting) while the same value for the control group was 72.3 ± 8.7 s (Figure 7D). There was also no difference between the amounts of time the 2 groups of animals spent with non-social behaviour (134.9 ± 15.5 s in treated vs. 150.4 ± 22.7 s in control).

#### 3.3.2. Locomotion, Anxiety- and Depression-like Behaviour after Htr2c Antagonist Treatment

The number of entries to the zones of the open-field area for the control and the treated animals were as follows: 3.0 ± 1.5 vs. 4.2 ± 1.5 times for the periphery; 3.4 ± 1.7 vs. 4.4 ± 1.6 times for the middle zone, and 0.4 ± 0.2 vs. 0.2 ± 0.2 times for the centre (Figure 7E). Thus, the locomotor activity measured in the open-field test did not differ between the two groups using *t*-tests.

In the elevated-plus-maze test, the control animals entered the open arms 13.0 ± 3.8 times and the closed arms 10.2 ± 3.3 times, while antagonist-treated animals entered the open arms 13.0 ± 2.2 times and the closed arms 13.2 ± 2.4 times. There was no significant difference between the two groups using *t*-tests (Figure 7F).

Depression-like behaviour was assessed using the forced-swim test. The control group spent 47.0 ± 3.4% of their time climbing, 29.2 ± 3.4% swimming, and 23.8 ± 6.5% floating. The group treated with the antagonist spent 39.4 ± 2.6% of their time climbing, 34.9 ± 5.1% swimming, and 25.6 ± 3.7% floating. There were no significant differences in any of the behavioural elements between the two groups using *t*-tests (Figure 7G).

## 4. Discussion

### 4.1. Behavioural Effects of Social Isolation

The study presents a detailed behavioural assessment of the effects of 10 days of social isolation on rats. The reduced time the isolated animals spent with a conspecific suggests their reduced sociability, which could have been even underestimated due to the more than optimal lightning during the three-chamber tests [65]. Furthermore, the isolated animals also showed no difference between the time spent with the novel and familiar conspecific in the social novelty discrimination test, suggesting impaired social recognition. Thus, the performance of isolated rats in these tests was impaired, similar to the results found in previous studies following both longer [66,67] but also shorter social isolation [68,69]. In turn, previously isolated animals demonstrated an increased amount of affiliative behaviour when they were allowed to freely interact with a conspecific. Previous studies using subchronic social isolation have confirmed that this condition increases social interactions [38,70] in mice and also in rats [71]. No specific behavioural element was found to have increased more than the others, suggesting that overall social motivation was elevated. This finding is consistent with the social homeostasis theory, which explains why social deprivation leads to increased social interactions when the opportunity arises [39,72]. Despite reduced interest in a separated conspecific in the sociability test, elevated social interactions occur. This could be explained by the fact that only direct contact can alleviate the increased homeostatic demand of the isolated animal, as previously demonstrated [38,73]. When investigating the timeline of the rebound in social interactions, it was discovered that social interactions gradually increase up to 5 days in mice, depending on the strain [38]. A rat study examined time points beyond 5 days of social isolation and found a peak of social rebound at 7 days [71]. In our study, we used 10 days of social isolation to ensure sufficient time for gene expression alterations to be established. The obtained behavioural data confirms that social interactions remain elevated at this time point. This is significant because prolonged social deprivation, such as six weeks of social isolation, can lead to behaviour resembling depression including reduced social interactions [21,22,23,24,26]. The results of unaltered responses in the elevated-plus-maze and the forced-swim tests in our study support the view that the animals do not exhibit signs of anxiety- and depression-like behaviour as compared the pair-housed animals. Since several tests were performed on the same days to restrict the duration of all behavioural experiments to 2 days in an attempt to be performed on the same or almost the same day as the dissections, all animals could have had elevated stress levels. However, a trend towards anxiety-like behaviour was found in the isolated animals, which may indicate the already initiated development of anxiety, as it develops within 30 days of social isolation [74]. Furthermore, it is possible that the anxiogenic responses were underestimated due to the dim light used in the elevated-plus-maze test [65].

No changes were found in the aggressive behaviour of the rats during the male-intruder test. Aggressive behaviour is characteristic of animals that have been socially deprived during development. Even during the post-weaning period, they exhibit increased aggression towards conspecifics [11,12,13]. Long-term social isolation in adults can also lead to increased aggression [19]. However, our study shows that 10 days of social isolation did not result in increased aggression in male rats. It is important to note that aggression typically appears 14 days after adult social isolation and gradually increases with prolonged isolation [19]. An increase in locomotor activity is suggested by the increased entry to all zones in the open-field test, which is consistent with previous studies on the effect of adult social isolation on locomotor activity in rats [75], while 7 weeks of social isolation reduces locomotor activity [76]. In addition, our data extend previous research on increased social interactions without examining aggressive or depressive behaviour during this period of social isolation [38,71]. Therefore, our battery of behavioural tests provides a more complete picture of the behaviour of short-term isolated male rats. The changes include decreased sociability and social recognition, increased social interactions, and hyperlocomotion, without any change in aggression, anxiety, or depressive-like behaviour. These results are very different from the effects of long-term isolation, which results in aggression, anxiety, or depression-like behaviour with reduced locomotion [11,12,13,16,17,18,21,22,23,24], but are similar to changes previously identified for 5–7 days of social isolation [38,68,69,70,71]. As we did not investigate the effect of isolation in females, our data do not provide information on possible sexual dimorphism. The previous literature has established that social rebound is present in both sexes, with only subtle differences found between them. For example, females may experience a slightly higher degree of hyperlocomotion after social deprivation [37] or somewhat more pronounced depression-like behaviour, pain sensitivity, and learning in females [77,78].

### 4.2. Social Isolation-Induced Gene Expressional Alterations

To investigate the gene expression changes underlying the behavioural changes induced by 10 days of social isolation, RNA-seq was performed in the mPFC, selected as a brain area responsible for the formation of social behaviour [79]. The consistently high mapping percentages to the reference genome suggest an excellent quality of the sequencing data. Using strict criteria, 46 genes showed altered expression levels, suggesting an effect of social isolation on the gene expression pattern of the mPFC. The number of downregulated genes (32) was higher than the number of upregulated genes (14). The symmetric volcano plot suggests a balanced alteration of genes towards up- and downregulation, arguing against a technical bias causing the higher number of downregulated genes, suggesting it is the result of social isolation-dependent brain alteration. Bioinformatic analysis of the differentially expressed genes (DEGs) and the pathways they participate in, including GO classification and KEGG functional enrichment, revealed that monoaminergic and peptidergic systems are affected by social isolation, and a major biological function they share is addiction. Localization of DEGs suggested that synapses may be most affected, both presynaptic terminals and postsynaptic targets. It is reasonable to propose that the altered genes underlie the behavioural changes measured following short-term social isolation. The finding that changes were found in monoaminergic and peptidergic systems is not surprising given the established role of these neurochemical systems in the mPFC in the control of social behaviour [28,29,40,41,42]. However, the actual pattern of changes is specific to short-term social isolation, as long-term social isolation also affected the expression of genes involved in depression, as well as genes whose protein products are located at synapses in the mPFC [22,50], but the altered genes showed marked differences in line with the different behaviour of the animals in the two situations. Based on our results, it cannot be determined if the changes we observed after 10 days of social elevation persist or not for long-term social deprivation, and consequently, whether they contribute to behaviour changes following long-term social deprivation.

The mPFC has been implicated in social behaviours and processing social information as well as the consequences of social isolation [29,30,80,81]. Socially isolated animals may exhibit alterations in mPFC activity, connectivity, and gene expression [82], leading to changes in the structure and function of the mPFC [34,35]. At the same time, social isolation is a significant risk factor for the development and maintenance of addiction in humans [83] as individuals lacking positive social connections and support systems might turn to drugs or other substances as a way to cope with loneliness, stress, or negative emotion [84]. The mPFC plays a critically important role in addiction in animal models, too [30,81] in line with its role in reward processing and decision-making [85]. The present study found that 10 days of social isolation alters the expression of genes in the mPFC involved in addiction suggesting the mPFC potentially contributes to the promotion of addiction by social isolation. Interestingly, the GABAergic system was not affected based on our results despite its involvement in the control of social behaviour by the mPFC [43,44,45]. It is possible that a limitation of the study, the pooling of different parts of the mPFC, most importantly the prelimbic and infralimbic cortices masked the changes because the GABAergic systems of these otherwise adjacent brain regions differ from each other structurally and functionally [86,87]. In addition to the monoaminergic system, neuropeptides, e.g., oxytocin and corticotropin-releasing hormone, which we did not find to be altered in the present study, might also be involved in the control of prefrontal control of social interactions [88,89]. Finally, another limitation of the present study is, that only the medial prefrontal cortex was investigated while other brain regions also involved in the control of social behaviour, such as the striatum or the medial preoptic area, have not been investigated.

Three genes related to dopaminergic, peptidergic, and serotonergic neurotransmission machinery, respectively, namely the regulator of G protein signalling 9 (Rgs9), prodynorphin (Pdyn), and serotonin 2C receptor (Htr2c), were validated using the RTqPCR technique. Their reduced expression levels following 10 days of social isolation were confirmed by the independent technique using dissections of mPFC from a new set of animals. This provides strong evidence for the validity of the observed gene expression changes. Thus, we have identified three genes that may contribute to the behavioural alterations induced by social isolation.

The Rgs genes encode proteins, which modulate signalling pathways initiated by G protein-coupled receptors [90]. RGS9 proteins are responsible for the rapid turnoff of G protein-coupled receptor signalling including μ-opioid receptor (MOR) [91] as well as dopamine signalling via the D2 receptor (DRD2) [92]. Rgs9 has 2 splice variants, Rgs9-1 in rods and cones of the retina contributes to recovery from bright flash while Rgs-2 regulates dopamine and opioid signalling in the brain [93] including the mPFC [94]. Based on single-cell sequencing data, Rsg9 is expressed in 2 types of layer VI excitatory neurons one projecting to the thalamus and the other to cortical regions while also present in some inhibitory somatostatin interneurons [95], suggesting that its level is reduced in some of these cells following social isolation. The Rgs9 was identified as a protein-protein interaction node of genes altered in mPFC of addicted mice suggesting its role in addiction [96]. While its biological function remains obscure, a genome-wide association study suggested its role in sweet food liking [97]. In turn, the present findings suggest its potential role in the control of social interactions.

The Pdyn gene encodes the protein precursor prodynorphin, which is then cleaved to produce the endogenous opioid peptide dynorphin (Dyn), which plays a role in pain perception, stress responses, reward processing, and other physiological processes in the central nervous system by acting on the kappa opioid receptors (KOR) [98,99]. The Dyn/KOR system is highly enriched in the mPFC [100,101]. Dyn is present in both pyramidal and inhibitory cell types in the mPFC [102]. Based on single-cell sequencing data [95], Pdyn is expressed in 2 different types of somatostatin interneurons and a layer V excitatory pyramidal neuron whose involvement in the behavioural changes following social isolation is suggested by the reduced Pdyn levels. Dyn acts on presynaptic KORs to suppress glutamate release from some afferent inputs to the mPFC. In addition, Dyn suppresses somatostatin interneuron-mediated inhibition. Thus, by acting on different circuit elements, Dyn/KOR signalling controls how different limbic inputs drive spiking in mPFC neurons [103]. As a potential behavioural effect, it was shown that local Dyn cells are activated by threats and the release of Dyn reduced defensive behaviours [104] and disrupted cognition [105]. While the cortical Dyn/KOR system has been implicated in the processing of motivationally-charged emotional information [101], its downregulation following social isolation suggests that a reduced functioning of the mPFC Dyn/KOR system contributed to the changes in social behaviours following social isolation. A previously described action of Dyn is suppression of social interaction [106], which effect can be potentially counteracted by its reduced level in the mPFC of socially isolated animals suggesting that reduced Pdyn might contribute to elevated social interaction induced by 10 days of social isolation.

### 4.3. The Role of Serotonin 2C Receptor in Social Novelty Discrimination

The serotonin (5HT—5-hydroxytryptamine) system of the brain participates in the control of a variety of different behaviours including emotional, sexual, aggressive, and other social behaviours as well as sleep and feeding behaviours [107,108,109]. Dysregulation of serotonin signalling has been implicated in mood disorders, including depression and anxiety; consequently, drugs acting on serotonin receptors have demonstrated a potential for modulating mood-related behaviours [110,111,112]. The serotonin 2C receptor gene (Htr2c) encodes the serotonin 2C receptor (5-HT2CR), a G-protein coupled receptor of serotonin. The Htr2c is part of the larger gene family encoding the 5HT2 receptors [113]. Htr2c is expressed in widespread brain regions [113,114] to affect various behaviours and moods [115]. Based on the present results, its reduced level in the mPFC following short-term isolation, shows the 5-HT2CR receptor may be involved in the control of the behavioural alterations induced by social isolation. A reduction in the expression level in the mPFC is not unique to social isolation. Htr2c expression was lower in the mPFC of rats with intense addictive behaviour, suggesting that it is also involved in addiction [116]. A downregulation of Htr2c expression was found in mPFC, which became significant after 3 weeks of antidepressant treatment [117]. A decreased expression of Htr2c in the mPFC of schizophrenia patients was also reported [118] suggesting that alterations in the expression level of Htr2c are a common regulatory mechanism in different adaptive situations.

Because of the clinical importance of the serotonergic system and the availability of drugs acting on its receptors including Htr2c, we functionally addressed the role of Htr2c in the behavioural alterations induced by social isolation. The purpose of this experiment was to establish if recovering the changes of monoaminergic signalling in PFC, which were induced by social isolation in adulthood, was possible by reversing the measured changes in the Htr2c level. Agomelatine as an antagonist at serotonin 2C receptors [53] was applied. In order to avoid daily injections, which may create stress interfering with social behaviour, a continuous injection of Agomelatin was applied using osmotic minipumps. The minipumps delivered a daily quantity of 0.08 mg/kg body weight Agomelatin. Estimating a 1 to 300 brain-to-body volume ratio, this intracerebroventricular dose corresponds to about 24 mg/kg systemically injected Agomelatine, which has been demonstrated to be the in vivo effective dose [119]. Injection of Agomelatine was effective in mimicking the effect of social isolation as far as reducing social novelty discrimination in non-isolated animals suggesting that this action of social isolation may be caused by the reduced functioning of 5HTR2c due to Htr2c expression level. Although Agomelatine also acts on melatonin receptors [120], the altered Htr2c but unchanged melatonin receptor levels argue against exerting its action via the melatonin receptors when abolishing social novelty discrimination. Other behavioural consequences of social isolation were not affected suggesting that those behavioural changes do not involve the reduced Htr2c functioning but supposedly are the results of changes in the expression of other genes. Furthermore, Agomelatine, despite being an antidepressant [121], did not alter the depression-like behaviour of the rats. It is in line with the fact, that the pair-housed rats were not depressed, and also that the development of antidepressant properties requires at least 14 days [119] while the testing of behaviour was performed 10 days after the onset of Agomelatine administration.

In the mPFC, Htr2c is expressed in layer V/VI near projecting, and in layer II as well as layer VI Car2 intratelencephalic excitatory neurons. In addition, it is also expressed in vasoactive intestinal peptide (VIP) and gamma-synuclein (Sncg) interneurons [95] suggesting that some of these cells may be involved in eliminating social novelty discrimination. Indeed, it has been previously reported that Htr2c may be involved in the control of social interactions because knock-in mice harbouring a human loss-of-function Htr2c variant developed reduced social exploratory behaviour [122].

## 5. Conclusions

This paper examines the relationship between social isolation and genes in the mPFC of rats. The effects of short-term isolation on behaviour are discussed and distinguished from the effects of long-term isolation but we caution the readers around over-generalization from this contrived model as we did not mimic naturally occurring conditions. Nevertheless, the results identify the monoaminergic and peptidergic systems that are affected and may play a role in the altered behaviour of the animals. Functional evidence for the role of Htr2C was provided for the reduced social novelty discrimination, as an antagonist of the serotonin receptor product mimicked the reduced social novelty discrimination caused by preceding social isolation in non-isolated animals. Understanding the role of the mPFC in the effects of social isolation could lead to the development of therapeutic interventions that ameliorate the negative consequences of social deprivation seen in some neuropsychiatric disorders, such as autism and social anxiety.

## Figures and Tables

**Table 1:** The information on RNA quality and mapping ratio for isolated (Isol) and control (Ctrl) groups in RNA-seq.

Sample	RNA Integrity Number (RIN)	Total Clean Reads (M)	Total Mapping Ratio (%)	Uniquely Mapping Ratio (%)	Total Gene Mapping Ratio (%)
Ctrl_1	9.4	41.8	96.7	93.4	48.3
Ctrl_2	9.5	39.3	96.2	86.9	45.3
Ctrl_3	9.6	34.4	97.7	93.5	51.8
Ctrl_4	8.3	33.9	96.6	92.6	34.1
Ctrl_5	10	35.6	97.4	92.4	49.3
Ctrl_6	10	36.7	97.4	92.7	51.5
Isol_1	8.3	32.2	96.6	92.8	33.7
Isol_2	8.9	32.7	97.4	93.6	44.5
Isol_3	9.3	39.2	97.5	93.3	51.9
Isol_4	9.6	37.7	96.4	92.0	50.4
Isol_5	9.7	31.3	97.7	93.0	56.5

**Table 2:** The table shows the downregulated and upregulated protein-coding genes in the sequencing experiment with gene id and name, protein name, and biological function. The table contains the value of the changes (fold change, *p*-value, *q*-value) for each gene.

Gene_ID	Gene	Protein Name	log2 of Fold Change	*p*-Value	*q*-Value	Biological Function	Regulation
MSTRG.19509, ENSRNOG00000011111	Cipc	CLOCK-interacting pacemaker	−6.47	5.0 × 10^−5^	0.02	exhibits circadian regulation in multiple tissues	down
MSTRG.700	N/A	N/A	−4.38	5.0 × 10^−5^	0.02	N/A	down
MSTRG.21449, ENSRNOG00000054065	N/A	N/A	−3.24	5.0 × 10^−5^	0.02	N/A	down
MSTRG.13085, ENSRNOG00000009577	Ndst4	N-deacetylase and N-sulfotransferase 4	−2.16	5.0 × 10^−5^	0.02	catalyzes the transfer of a sulfate group from 3′-phosphoadenosine 5′-phosphosulfate to the hydroxyl group of an acceptor	down
MSTRG.13636, ENSRNOG00000001302	Adora2a	adenosine A2a receptor	−1.81	5.0 × 10^−5^	0.02	receptor for adenosine	down
MSTRG.106, ENSRNOG00000012311	Slc35d3	solute carrier family 35, member D3	−1.78	5.0 × 10^−5^	0.02	carbohydrate transport and pyrimidine nucleotide-sugar transmembrane transport	down
MSTRG.4488, ENSRNOG00000003800	Rgs9	regulator of G-protein signalling 9	−1.65	5.0 × 10^−5^	0.02	inhibits signal transduction b	down
MSTRG.19469, ENSRNOG00000027645	Syndig1l	synapse differentiation-inducing 1-like	−1.61	5.0 × 10^−5^	0.02	predicted to be an integral component of the membrane	down
MSTRG.13873, ENSRNOG00000049580	Gpr6	G protein-coupled receptor 6	−1.43	5.0 × 10^−5^	0.02	activate cyclic AMP, promotes neurite outgrowth and blocks myelin inhibition in neurons	down
MSTRG.21857, ENSRNOG00000008428	Drd2	dopamine receptor D2	−1.4	5.0 × 10^−5^	0.02	mediated by G proteins which inhibit adenylyl cyclase	down
MSTRG.17137, ENSRNOG00000007647	Oprk1	opioid receptor, kappa 1	−1.38	5.0 × 10^−5^	0.02	receptor for endogenous alpha-neoendorphins and dynorphins	down
MSTRG.2540, ENSRNOG00000030180	Lrrc10b	leucine-rich repeat containing 10B	−1.33	5.0 × 10^−5^	0.02	required for endogenous cardiac regeneration	down
MSTRG.17163, ENSRNOG00000008943	Penk	proenkephalin	−1.27	5.0 × 10^−5^	0.02	increases glutamate release and decreases GABA concentration	down
MSTRG.21754, ENSRNOG00000035548	Mir3596a	microRNA 3596a	−1.27	5.0 × 10^−5^	0.02	N/A	down
MSTRG.19142, ENSRNOG00000005286	Coch	cochlin	−1.11	5.0 × 10^−5^	0.02	plays a role in the control of cell shape and motility in the trabecular meshwork	down
MSTRG.21176, ENSRNOG00000041492	Mir1249	microRNA 1249	−1.11	5.0 × 10^−5^	0.02	regulatory role in inflammation, tumor progression, and cell differentiation	down
MSTRG.16758, ENSRNOG00000012876	Slc6a13	sodium- and chloride-dependent GABA transporter 2	−1.08	5.0 × 10^−5^	0.02	mediates sodium- and chloride-dependent transport of GABA	down
MSTRG.12399, ENSRNOG00000014793	Gpr149	G protein-coupled receptor 149	−1.07	1.0 × 10^−4^	0.04	chemical synaptic transmission, G-protein coupled receptor signalling pathway, and neuropeptide signalling pathway	down
MSTRG.9545, ENSRNOG00000023688	Drd1	dopamine receptor D1	−1.05	5.0 × 10^−5^	0.02	encodes the D1 subtype of the dopamine receptor	down
MSTRG.14132	N/A	N/A	−1.03	5.0 × 10^−5^	0.02	N/A	down
MSTRG.23800	N/A	N/A	−0.92	1.0 × 10^−4^	0.04	N/A	down
MSTRG.2495, ENSRNOG00000054315	Snord27	small nucleolar RNA, C/D box 27	−0.91	5.0 × 10^−5^	0.02	conversion of one or more primary RNA transcripts into one or more mature RNA molecules	down
MSTRG.7907, ENSRNOG00000024061	Rarb	retinoic acid receptor, beta	−0.89	5.0 × 10^−5^	0.02	binds retinoic acid, the biologically active form of vitamin A	down
MSTRG.8731, ENSRNOG00000013330	Cdhr1	cadherin-related family member 1	−0.89	5.0 × 10^−5^	0.02	it is a photoreceptor-specific cadherin that plays a role in outer segment disc morphogenesis	down
MSTRG.16061, ENSRNOG00000011184	Slc13a4	sulphate transporter 1	−0.87	5.0 × 10^−5^	0.02	predicted to enable sodium:sulfate symporter activity and involved in anion transmembrane transport	down
MSTRG.13986, ENSRNOG00000015550	Ptgds	prostaglandin D2 synthase	−0.82	5.0 × 10^−5^	0.02	catalyzes the conversion of PGH2 to PGD2	down
MSTRG.15056, ENSRNOG00000026036	Pdyn	prodynorphin	−0.81	5.0 × 10^−5^	0.02	Leu-enkephalins compete with and mimics the effects of opiate drugs	down
MSTRG.23424, ENSRNOG00000016957	Igfbp2	insulin-like growth factor binding protein 2	−0.77	5.0 × 10^−5^	0.02	inhibits IGF-mediated growth and developmental rates	down
MSTRG.15889, ENSRNOG00000007374	Tac1	tachykinin, precursor 1	−0.76	5.0 × 10^−5^	0.02	a neuropeptide, which excites neurons to evoke behavioural responses	down
MSTRG.24554, ENSRNOG00000030877	Htr2c	5-hydroxytryptamine receptor 2C	−0.75	5.0 × 10^−5^	0.02	G-protein coupled receptor for 5-hydroxytryptamine	down
MSTRG.4265, ENSRNOG00000028404	Ppp1r1b	protein phosphatase 1, regulatory subunit 1B	−0.74	5.0 × 10^−5^	0.02	inhibitor of protein-phosphatase 1	down
MSTRG.11897	N/A	N/A	−0.71	1.0 × 10^−4^	0.04	N/A	down
MSTRG.16148, ENSRNOG00000006228	Pdia4	protein disulfide isomerase family A, member 4	7.68	5.0 × 10^−5^	0.02	a member of the disulfide isomerase family of endoplasmic reticulum proteins that catalyze protein folding and thiol-disulfide interchange reactions	up
MSTRG.14025, ENSRNOG00000028021	Rps13	ribosomal protein S13	4.11	5.0 × 10^−5^	0.02	component of the small ribosomal subunit	up
MSTRG.19735, ENSRNOG00000035600	Mir154	microRNA 154	3.24	5.0 × 10^−5^	0.02	inhibits cell proliferation, migration, and invasion	up
MSTRG.22465, ENSRNOG00000030714	Bsn	bassoon	3.04	5.0 × 10^−5^	0.02	involved in organizing the presynaptic cytoskeleton	up
MSTRG.3480, ENSRNOG00000032825	Rpl30	ribosomal protein L30	1.91	5.0 × 10^−5^	0.02	component of the large ribosomal subunit	up
MSTRG.10334, ENSRNOG00000056958	Snora21	small nucleolar RNA, H/ACA box 21	1.89	5.0 × 10^−5^	0.02	plays an important role in cancer progression	up
MSTRG.23546	N/A	N/A	1.47	5.0 × 10^−5^	0.02	N/A	up
MSTRG.9257	N/A	N/A	1.21	5.0 × 10^−5^	0.02	N/A	up
MSTRG.16897, ENSRNOG00000007830	Apold1	apolipoprotein L domain containing 1	1.01	5.0 × 10^−5^	0.02	plays a role in the regulation of endothelial cell signalling and vascular function	up
MSTRG.20900, ENSRNOG00000043465	Arc	activity-regulated cytoskeleton-associated protein	0.87	5.0 × 10^−5^	0.02	master regulator of synaptic plasticity	up
MSTRG.21400, ENSRNOG00000007607	Nr4a1	nuclear receptor subfamily 4, group A, member 1	0.85	5.0 × 10^−5^	0.02	orphan nuclear receptor	up
MSTRG.3306, ENSRNOG00000003977	Dusp1	Dual specificity protein	0.79	5.0 × 10^−5^	0.02	dual specificity phosphatase	up
MSTRG.20616, ENSRNOG00000049283	N/A	N/A	0.64	5.0 × 10^−5^	0.02	N/A	up
MSTRG.14769, ENSRNOG00000009549	Fbxo3	F-box protein 3	0.57	5.0 × 10^−5^	0.02	promotes the proteasomal degradation of Smurf1	up

## Data Availability

The raw sequencing data files are available in the Sequence Read Archive (SRA) at the National Center for Biotechnology Information (NCBI) under the Bioproject ID: PRJNA1100307.
